# Salmonella infection in chickens: pathogen, pathogenesis, and dietary non-drug feed additives as alternatives to antibiotics — a comprehensive review

**DOI:** 10.1007/s12223-025-01403-z

**Published:** 2026-02-28

**Authors:** Nahed A. El-Shall, Mehmet Cemal Adiguzel, Wafaa A. Abd El-Ghany, Walaa A. Gamal

**Affiliations:** 1 Department of Poultry and Fish Diseases, Faculty of Veterinary Medicine, Alexandria University, Alexandria, 21944 Egypt; 2 Department of Microbiology, Faculty of Veterinary Medicine, Ataturk University, Erzurum, Turkey; 3 Poultry Diseases Department, Faculty of Veterinary Medicine, Cairo University, Giza, 12211 Egypt; 4 Eldoctor Veterinary Clinics, Alexandria, 23625 Egypt

**Keywords:** Salmonella, Antibiotics, Probiotics, Nanoparticles, Bacteriophages, Chickens

## Abstract

*Salmonella* infections in poultry can result in systemic, localized diseases, or prolonged asymptomatic carriers. Avian host-specific *Salmonellae* such as *S. Pullorum* and *S. Gallinarum* can spread vertically or horizontally from diseased birds or contaminated feed, water, or litter (fecal-oral route), resulting in significant mortality rates of up to 80% in chicks and poults during 1st 2–3 weeks of age. Furthermore, one of the most common food-borne diseases in humans is caused by Paratyphoid *Salmonellae*. The antibiotic overuse or misuse in animal production raises concerns due to the formation of resistant bacteria or genes and tissue residues. Currently, *Salmonellae* are among the most antibiotic-resistant bacteria and cause the most severe infections in both human and animals. Besides biosecurity, the inclusion of feed additives such as probiotics and their derivavtives, phytobiotics, nanoparticles, and egg yolk immunoglobulins can help to lessen the burden of *Salmonella* infections (colonization and shedding) in poultry and enhance health and immune response. Additionally, bacteriophages, the bacteria-specific viruses, are considered a biocontrol measure for Salmonella depending on using the appropriate phage, doses, time, and route of administration. These interventions have the potential to replace conventional antibiotics in therapeutic settings by exhibiting bactericidal effects without the risk of emerging antibiotic-resistant strains or intestinal dysbiosis. Moreover, the synergistic interactions between these interventions can provide a more inclusive, efficient, and integrated approach for managing Salmonellosis in poultry flocks. This current review aims to spotlight on the avian *Salmonella* infections and the role of antibiotic alternatives to control it.

## Introduction

Poultry species are highly susceptible to various important bacterial diseases including Salmonellosis. It has been widely distributed all over the world with variable incidences and has a significant importance for both poultry industry and humans’ health over 125 years. In poultry, *Salmonella* infections are usually associated with drastic drop in the performance indices (Lamichhane et al. 2024). *Salmonella* spp. are Gram-negative facultative anaerobic bacilli, family *Enterobacteriaceae*. *S. Pullorum* and *S. Gallinarum* are non-motile avian-host-specific biovars of *S. eneterica subspecies enterica serovar Gallinarum* causing pullorum (chicks and poults) and fowl typhoid diseases (mature birds), respectively. Furthermore, the motile *Paratyphoid Salmonellae* (non-typhoid) can infect a broad range of hosts, including people, domestic and wild animals and birds (Abd El-Ghany 2020a; CDC, 2025). The non-typhoidal *Salmonella* spp. involve *S. Enteritidis*, *S. Typhimurium*, *S. Kentucky*, *S. Infantis*, *S. Newport*, *S. Montevideo*, *S. Thompson*, *S. Javiana*, *S. Heidelberg*, *S. Muenchen*, *S. Braenderup*, and *S. Saintpaul* (Andino and Hanning 2015). The European Union identified *S. Eenteritidis* and *S. Ttyphimurium* as the second most frequently reported foodborne intestinal infections in human (EFSA, 2021). Galliforms including domestic chicken, turkeys, water fowl, and other poultry species are susceptible to clinical *Salmonella* infections (Ramtahal et al. 2022). *Salmonella*-infected chicks show inappetence, pasty whitish diarrhea, omphalitis, dehydration, growth retardation, lameness, and blindness, while adults may show drop in egg production and hatchability parameters (Sania et al. 2022). The mortality rate may reach to 80% in baby chicks, however, older ages over 3-weeks-old rarely show mortality (Gast and Beard 1992).

The antibiotic therapy is commonly recommended for counteracting *Salmonella* infection threats in the poultry (Suresh et al. 2018). The used antimicrobials should follow strict guidelines and antibacterial susceptibility surveillance tests to identify the updates of *Salmonellae* resistance (Nassar et al. 2023). Despite the rapid and significant efficacy of antimicrobials in poultry farms, their uncontrolled usage may diminish their efficiency, induce tissue residues (Lima et al. 2023) and environmental contamination (Mohammadi et al. 2020), and develop multi-drug resistance (MDR) **(**Chen et al. 2020a; Jibril et al. 2021). The developed MDR contributes to the selective pressure and transfer of resistance genes among the bacterial strains. An approximately 200,000 people in the USA had been died annually from antibiotic-resistant infections (Kadri 2020). The isolated *Salmonella* spp. strains from animal food sources exhibited high levels of resistance to the commonly used antibiotics in veterinary medicine (Abd El-Aziz et al. 2021). This highlights the importance of implementing safe control strategies in poultry production systems. Numerus dietary additive alternatives have been proposed to circumvent the problems associated with the hazard use of antibiotics in poultry. Particular attention has been paid to investigate the potential advantages of phytobiotics, direct feed microbials and their derivatives, egg yolk immunoglobulin Y, nanoparticles, and bacteriophage therapy (El-Kafrawy et al. 2023; Nassar et al. 2025; Clavijo et al. 2019; Thanki et al. 2023; El-Shall et al. 2019, 2024; Alagawany et al. 2025; Abd El-Ghany 2020b, 2025; Abd El-Hack et al. 2022). They have been used to reduce the incidence of avian Salmonella infections, maintaining optimal gut health, and enhancing the immune system. Therefore, this comprehensive literature review article aims to spotlight on *Salmonella* epidemiology, antibiotic resistance, and synthesize the current understanding of the role of some dietary interventions in mitigating the burden of *Salmonella* infections in poultry.

## Avian *Salmonella* spp. Epidemiology and clinical signs

*Salmonella’s* infections are caused by Gram-negative, rod-shaped, and facultative anaerobic bacteria, genus *Salmonella*, family *Enterobacteriaceae*. Both *S. Enterica* and *S. Bongori* are the two species into which all *Salmonellae* are divided. Six subspecies of *S. eneterica* were identified, including *S. eneterica subspecies enetrica* and *S. eneterica subspecies Arizonae*. The serological identification of *S. eneterica* revealed the presence of 51 serogroups and 2600 antigenically different pathogenic serovars for animals and human (Zou et al. 2016). *S. eneterica subspecies enetrica* include the host-adapted *S. Gallinarum* and *S. Pullorum* and non-host adapted Paratyphoid *Salmonellae*. Paratyphoid *Salmonellae* primarily cause foodborne illness in human through the ingestion of contaminated eggs and meat products. The most prevalent Paratyphoid serovars are *S. Enteritidis* and *S. Typhimurium* (Shaji et al. 2023), but they rarely induce acute clinical illness of extremely vulnerable young birds under stressful circumstances (Gast and Porter 2020). Moreover, *S. Arizonae*, a motile and non-host specific member of the *S. enterica subspecies arizonae*, primarily affects young turkeys causing arizonosis. It causes neurological disorders such as paralysis, tortricolus, and opisthotonus, in addition to blindness and increased mortality of poults. Breeders have *S. Arizonae* may show reduced production and hatchability and a death rate of up to 50% (Gast and Porter 2020).

*Salmonella* spp. may survive in soil and water for weeks to months and can adapt to a variety of environments (Schierstaedt et al. 2020; Talukder et al. 2023). The transmission of *Salmonellae* may occur either horizontally or veritically (El-Sharkawy et al. 2017). The contact with droppings and contaminated feed, water, and litter of infected or chronic carries is the common and primary horizontal spreading route of *Salmonellae* in poultry flocks (Berhanu and Fulasa 2020). Skin wounds and contact with reproductive secretions, blood, and contaminated equipments play also a key role in its transmission. In addition, *Salmonella* spp. can infect the ovaries and oviduct through ascending transmission from cloaca or descending spread via the bloodstream, causing vertical infections of embryos. Infected birds or carriers may shed the organism in their droppings causing egg shell contamination after laying (Liu et al. 2022). Accordingly, the hatched chicks from infected breeders become long-term *Salmonella* carriers (Berhanu and Fulasa 2020). The mechanical spread through personnel, rodents, insects, and pets plays an important role for the rapid transmission of such infection (Liljebjelke et al. 2005). Free living wild birds are regarded as the main reservoir and shedder for *Salmonellae* (Tizard 2004). Moreover, the contaminated animal byproducts (bone, meat, and feathers meals) are common factors for the lateral spread of Paratyphoid *Salmonella* (Crump et al. 2002).

 The pathogenicity of *Salmonella* spp. depends on some pathogen’s virulence factors, bird’s health status, and surrounding environment. Capsule, flagella, plasmids, adhesion systems, toxins, and type 3 secretion systems play important role in the host’s invasion, survival, and multiplication of *Salmonellae* (Boko et al. 2013). *Salmonella* pathogenicity island types 1 and 2 include the main virulence genes associated with the bacterium pathogenesis in chicken (Shaji et al. 2023). Intestinal inflammation is an additional factor that influences the rapid growth and spread of *Salmonella* spp. in the hosts. For instance, coccidial infection decreased the levels of *Salmonella *inhibiting volatile fatty acids in the intestine (Bjerrum et al. 2006). Besides, prior *Eimeria tenella* infection increased *Salmonella* intestinal colonization (Qin et al. 1995). Moreover, *Salmonella* spp. infections are exacerbated by immunological suppression associated with infectious bursal disease, reticuloendotheliosis viruses, or *Corynebacterium parvum* (Wyeth 1975; Motha et al., 1983; Corrier and Ziprin 1989). As well, fecal shedding and organ colonization by *S. Enteritidis* were considerably greater in chickens co-infected with avian influenza H9N2 virus (Arafat et al. 2020).

*Salmonella* infection is often started by ingestion of the bacterium, followed by colonization and penetration of the intestinal mucosal epithelium which resulted in systemic infection and colonization of spleen and liver (Henderson et al. 1999). After that, there is an immune evasion phase that helps in the clearance of *Salmonella* by producing antibodies and promoting T cell proliferation. Consequently, the pathogen may be cleared, the birds may die, or the pathogen may be partially cleared resulting in a carrier state (Revolledo and Ferreira 2012).

Salmonella infections in chicken, whether Paratyphoid or Pullorum, can cause dehydration, weakness, weight loss, watery feces, low appetite, increased morbidity and mortality in 2–3 weeks-old chicks (Gast and Porter 2020). Older birds exhibit infrequent signs and less acute course with arthritis. Significant morbidity and mortality can occasionally result from *S. Gallinarum* infections of mature birds. Malformed, discolored, and/or shrunken ovaries are the most common gross lesions. In addition, egg peritonitis and ascites may be observed in layers (Swayne 2020). In vertically infected flocks, it is possible to find dead and dying chicks shortly after hatching. Any organ can get a focal infection, including the kidney, heart, joints, meninges, and periosteum. Un-absorbed yolk sacs containing blood or flocculent, watery yellow fluid are frequently observed in baby chicks. Congested liver (bronzy) with white, pinhead-sized necrotic foci and enlarged spleen may be seen. The pericardium may thicken, and the pericardial sac may swell with turbid fluid (Barrow and Freitas Neto 2011). In the later stages of infection, typhlitis and cecal cores (white to yellow caseous material) are seen (Gast and Porter 2020).

### Antibiotic drug resistance

The prophylactic and therapeutic drugs such as sulfonamides, nitrofurans, chloramphenicol, tetracyclines, and aminoglycosides have been found helpful in reducing mortality and *S. Enteritidis’* fecal shedding in chickens. Chloramphenicol, nitrofurans, ampicillin and new generation cephalosporin sets are indicated in systemic salmonellosis (Ekperigin et al. 1983; Kabir 2010). However, no single drug or even drug combination has been found capable for complete elimination of infection from the treated flocks (EFSA, 2017). Thus, drugs can not eleminate the carrier status of the individual bird or flock as a whole. Moreover, indiscriminate use of antibiotics has resulted in the emergence of MDR *Salmonellae* (Kabir 2010; Gast and Porter 2020). Moreover, aimless use of antibiotics, including incorporation into feed as additives, usage for egg dipping, and routine therapeutic usage have also lead to increasing the prevalence of MDR strains (Ekperigin et al. 1983; Menghistu et al. 2012; Bupasha et al., 2020). Food borne pathogens including *Paratyphoid Salmonellae* are gaining serious importance and concern owing to the problem of MDR (Donado-Godoy et al. 2012; Li et al. 2013). It is estimated that 10 million deaths may occur by 2050 due to MDR threat (Talukder et al. 2023). Moreover, the World Bank has estimated that the global gross domestic product may fall by 1.1–3.8% by 2050 due to the independent impacts of MDR (Medina et al. 2020). While antibiotic resistance may occur naturally, the emergence of most resistant organisms is attributed to the acquisition of resistance genes through mutations, plasmid transfers, or existing natural resistance genes followed by a selective pressure (Ramirez-Hernandez et al. 2021; Song et al. 2024). The conserved zones in the accessory region of the *Salmonella* genome (genomic islands) as well as the mobile elements such as Class 1 integrons are closely linked to the bacterial resistance to antimicrobials (Partridge et al. 2018). Plasmids in *Salmonella* play a major role for the transfer of antibiotic resistance genes form one pathogen to another one. This transfer is possible between *Salmonella* spp. and the other enteric pathogens (Fricke et al. 2009) that usually occurs during conjugation. Transduction and transformation are the other possible ways by which the antibiotic resistance is transmitted between various food-borne pathogens (Okolo 1986). Therefore, it is required to track these resistance determinants and create practical controls for antibiotic resistance in order to track its human dissemination (Zahoor et al. 2024).

Various avian *Salmonella* strains are resistant to major antibiotics such as oxytetracycline, penicillin, and amoxicillin (St Amand et al. 2013), ampicillin and erythromycin (Shalaby et al. 2022), ampicillin, sulfamethoxazole-trimethoprim, tetracycline, nalidixic acid, chloramphenicol, and ceftriaxone (Bupasha et al., 2020), cephalosporin (Medeiros et al. 2011), fluroquinolones (Parveen et al. 2007), erythromycin and chloramphenicol (Ibrahim et al. 2021a), sulfamethoxazole and ceftiofur (Moraes et al. 2024), streptomycin (Kagambega et al. 2012), trimethoprim-sulfamethoxazole and nitrofurantoin (Vélez et al. 2017), etc. The emergence of MDR *Salmonella* strains was reported in most countries world-wide. For example, 72.72% of the tested *S. enterica* isolates were multi-drug resistant to at least 5 antibiotics; ceftriaxone, neomycin, sulphonamides, ampicillin, and oxytetracycline in Brazil (Taddese et al. 2019). All the isolated *Salmonella* strains (36.54%) from broiler chickens in Egypt were resistant to at least two antibiotics; ampicillin, erythromycin, streptomycin, gentamicin, doxycycline, colistin, and lincomycin (Shalaby et al. 2022). In china, *Salmonella* strains from broilers and poultry workers displayed MDR phenotypes and were resistant to 16 commonly used antibiotics such as ampicillin, streptomycin, kanamycin, amikacin, tetracycline, nalidixic acid, ciprofloxacin, trimethoprim/sulfamethoxazole, and chloramphenicol (Gong et al. 2019). The detected *Salmonella* isolates from organic and inorganic USA chickens were frequently resistant to at least one antibiotic (91.24%) or MDR (45.54%) (Punchihewage-Don et al. 2024). One of the interventions hypothesized to diminish the rhythm of drug resistance development is limiting the antibiotic usage for non-therapeutic purposes with sub-optimal doses. Randall et al. (2006) suggested maximization of the drug dose in a short duration for the treatment of *S. Typhimurium*. They used 2.5 times the recommended dose of enrofloxacin for 2-day regimen rather than 5 days, and thus minimizing the selection of drug resistance. Nevertheless, the worldwide trend to replace antibiotics by other alternative stratigies to control avian salmonellosis are discussed here.

### Non-drug feed additives for Salmonellosis control

In poultry systems, feed additives nutritional supplements have become extremely important due to their incredibly wide range of positive effects such as improving growth, production, and health, and strengthing immune system, while reducing diseases and mortalities (Fig. 1). These effects ensure a notable improvement in the animals’ quality of life and significantly improved the poultry industry economics (Rafeeq et al. 2023; Alagawany et al. 2025).

### Phytobiotics

Phytobiotics are broad subset of plant-derived bioactive compounds including alkaloids, phytosterols, carotenoids, as well as phenolic, nitrogen-containing, and organosulfur compounds that have variable effects on poultry production (Qin and Hou 2017). Phytobiotics are used as natural feed additive to subsitute synthetic antibiotics to promote growth, modulte immune response, and reduce the microbial and oxidative stress load (Zhang et al. 2017; Kikusato et al. 2021; Reda et al. 2024; El-Shall et al. 2020; Obianwuna et al. 2024).

The early in vitro study of Devi et al. (2010) demonstrated that treatment with eugenol at their minimal inhibitory concentration (0.0125%) and minimal bactericidal concentration (0.025%) resulted in complete inhibition of *S. Typhi* within 60 min exposure. Iwiński et al. (2022) demonstrated that a phytogenic blend comprising thymol, menthol, linalool, trans-anethole, methyl salicylate, 1,8-cineole, and p-cymene was effective in inhibiting antibiotic-resistant S. *Enteritidis*,* S. Typhimurium*, and *S. Kkentucky*. Additionally, a combination of thymol and carvacrol essential oils (EOs) significantly suppressed the growth and biofilm formation of pathogenic avian *E. Coli* O78 and *S. Pullorum* (Ding et al. 2022). The EOs derived from *Pulicaria Gnaphalodes* displayed strong antibacterial properties against *S. Typhimurium*, *E. Coli*, and *Mycobacterium Tuberculosis* (Gandomi et al. 2015). Kollanoor-Johny et al. (2010) found that using a modified cecal medium, several EOs compounds; trans-cinnamaldehyde, eugenol, thymol, and carvacrol were very effective against *S. Enteritidis* and *Campylobacter Jejuni* (*C. Jejuni*). Dos Santos et al. (2023) showed that the ethanol and ethyl acetate of *Cinnamomum Amoenum* extracts have an inhibitory effect against the growth of various avian *Salmonella* serotypes. In vivo, the different effects of phytobiotics on *Salmonella-*infected broiler chickens are illustrated in Table 1.

**Table 1:** The different effects of phytobiotics on *Salmonella*-infected chickens

Phytobiotics	Birds/Salmonella challenge	Experiment	Effects	Reference
EOs of thymol (A) A blend of EOs consisted of thyme, anise, oregano, carvacol, yucca extract, cinnamaldehyde, and some minor herbs (B).	Broilers/*S*. T*yphimurium*	EOs (A) (0.45 g/kg) + *S. Typhimurium* challenge EOs (B) (5 g/kg) + challenge at 1 day old.	Reduced *Salmonella* shedding rate.	Abudabos et al. 2016
Turmeric and garlic extract.	Broilers/*S. Pullorum.*	2.50% turmeric extract 2.00% garlic extract 2.50% garlic extract + challenge at 21 days old.	Significant reduction in inflammatory nodules and congestion and haemorrhages in liver and small intestine.	Purwanti et al. 2019
A herbal blend (HB) powder of *Astragalus*, *Panax notoginseng*, and licorice	Dwarf male chicks */S. Pullorum*	One-day-old chicks infected with *S. Pullorum* on day 1 and fed HB orally from days 4 to 10.	An improved growth performance, boosted immune function, promoted thymus and bursa development, and controlled intestinal flora after herbal treatment.	Wang et al. 2021
Intebio^®^ (wheat bran with a mixture of garlic, lemon, thyme, and eucalyptus essential oils).	Broilers/*S. Enteritidis.*	90 g of Intebio/ton of feed + challenge at 19 days old.	Enhanced immune response via upregulation of *AvBD10*, *IL6*, *IL8L2*, *CASP6*, and *IRF7* genes. Suppression of inflammatory reaction by reducing expression of the previous genes. Reduction of ceacal microflora biodiversity and dysbiotic disorders.	Laptev et al. 2019
Intebio^®^ (garlic, lemon, thyme, and eucalyptus essential oils).	Layers/*S. Enteritidis.*	Birds (aged 346 days) fed a diet supplemented with Intebio^®^ once a day (90 g/ ton of feed) + challenge at 367 days old.	The additive improved performance, degree of metabolism, and gastrointestinal microbiota.	Laptev et al. 2021
Allium-based phytobiotic.	21-week-old Layers/*S. Gallinarum biovar Pullorum ERG19.*	Phytobiotic (100 g/ ton of feed) + challenge at 21-week old.	The number of eggs laid and the FCR increased as a result of phytobiotic, but the internal quality of the eggs, and eggshell strength and thickness remained same. İt increased the prevalnece of Firmicutes and Bacteroidetes but reduced Proteobacteria and Actinobacteria phyla.	Ruesga-Gutiérrez et al. 2022
Powder and liquid mix of 7 plant extracts: oregano, eucalyptus, thyme, garlic, lemon, rosemary, and sweet orange. Sangrovit Extra^®^ is an herbal extract of plume poppy.	Broilers/*S*. *Typhimurium.*	Chicken groups received powder mix at (0.2 g/kg), liquid mix at (0.25 mL/L), and Sangrovit Extra^®^ at (0.15 g/kg) in the feed + challenge at 15 days old.	Powder mix and Sangrovit Extra^®^ significantly improved FCR and production efficiency factor during finisher period (15–35 d) to be comparable to antibiotic (avilamycin) and negative control groups.Three addiitves improved meat characteristics.	Aljumaah et al. 2020b
Blend (wheat germ powder, hops, and grape seed extract).	Broilers/no challenge.	Blend (wheat germ powder, hops, and grape seed extract (0.05%, 0.1% or 0.2% Magnolol at 100–400 mg/kg).	Increased *Lactobacillus*, but reduced *E. Coli* and *Salmonella.*	Zou et al. 2023
Pulicaria jauberti.	Broilers/no challenge.	*Pulicaria jauberti* powder (0, 3, 6, and 9 g/kg).	Increased *Lactobacillus*, but reduced *Salmonella* counts	Alharthi et al. 2023
Fenugreek (Trigonella foenum graecum). Turmeric (Curcuma longa L).	Domestic chickens (mixed infection with *S. Enteritidis* and *E.Coli.*	Chickens fed 1 kg/T of Fenugreek or Turmeric and were infectedorally on day 3 of age.	Fenugreek reduced cloacal salmonella shedding like enrofloxacin-treated group and lower than Turmeric. Fenugreek induced the best villus length and crypt depth.	Kerek et al. 2023
Organic acidmixture of formic acid, propionic acid. Carvacrol essential oil.	Broilers/no challenge	Organic acids mixture, (200 mg/kg), carvacrol essential oil (150 mg/kg), and a blend of organic acids and essential oil.	Increased *Lactobacillus*, but reduced *E.Coli*, *Salmonella*, and *Clostridium Perfringens* counts.	Islam et al. 2024
Thyme oil and Thyme oil-loaded-chitosan-nanoparticles.	Broilers/*S. Typhimurium.*	Thyme oil (1 ml/4L of the drinking water). Thyme oil-loaded-chitosan-nanoparticles (2 ml/chick) + challenge at 14 day old.	Reducing the shedding rate of *S. Typhimurium.*	Nassar et al. 2025

Additionally, Johny et al. (2010) found that plant-derived compounds increased the sensitivity of multidrug-resistant *S. Typhimurium* (DT104) to antibiotics. For instance, carvacrol heightened the pathogen’s vulnerability to sulfamethoxazole. Thymol and trans-cinnamaldehyde together made *Salmonella* more vulnerable to tetracycline, streptomycin, chloramphenicol, and sulfamethoxazole. When trans-cinnamaldehyde, β-resorcylic acid, carvacrol, thymol, and eugenol were combined, the pathogen became more susceptible to the tested antibiotics. The antimicrobial resistance may be lessened by strengthening the immune system since the bacterium will encounter several obstacles during colonization and infection (Qadri et al. 2021).

Regarding the immune-potentiating effect of phytobiotics, they could boost the host’s immune response and increase the bird’s resistance to *Salmonella* spp. infections. Phytochemicals may stimulate proinflammatory cytokines production, increase macrophages and natural killer cells activities, and enhance production of antibodies against *Salmonella* and antimicrobial peptides (Kogut et al., 2009; Norajit and Ryu 2012; Santos et al. 2019).

Phytobiotics can be broken down by gut microorganisms into simpler easily absorbed bioavailable metabolites which have good intestinal health-promoting benefits. Therefore, phytobiotics may increase the existence of beneficial bacteria (e.g. *Streptococcus* spp.), which generates the antimicrobial hydrogen peroxide or bacteriocins (Liu et al. 2023). Morover, phytobiotics have demonstrated promise in reducing microbial load especially *Salmonella* spp. owing to their richness in EOs, phenolic compounds, and saponins that can inhibit the bacterial development, survival, and replication (Almuzaini 2023). For instance, a phytogenic mixture (wheat germ, hops, and grape seed extract) displays synergistic antibacterial effects through its contents of flavonoids, tannins, xanthohumol, lectins, and β-acids (Zou et al. 2023). The bacterial cell membrane permeability and homeostasis are compromised by linalyl acetate and linalool in lavender EOs, which results in cell death (Liu et al. 2018; Farahat et al. 2021). It has been reported that the lipophilic characteristics of EOs permit them to invade the bacterial cell walls and mitochondria, disturbe and compromise the bacterial structure, increase permeability, and prevent bacterial invasion, reviewed in (Burt 2004; Yilmaz and Gul 2024). Moreover, EOs have the ability to inhibit the production of virulence factors in *Salmonella* pathogen (Zhang et al. 2019). EOs can alter the pH of the intestinal environment, hinder adhesion of microbes to epithelial surfaces, decrease toxin production, inhibit bacterial proliferation, and suppress biofilm formation (Zeng et al. 2015). Wang et al. (2023) demonstrated a suppression of *S. Enteritidis* growth by thymol, carvacrol, and cinnamon aldehyde due to lowering of gut pH, moisture content, and spoiling.

Therefore, the antimicrobial effects of phytobiotics are extensive and complex. The phytobiotics are able to increase the abundance of beneficial microbes which aids in the competitive exclusion of bacteria, improve nutrient metabolism, promote immune response, and decrease pathogen load suggests their capability to replace antibiotics and regulate microbiota-host relationship.

### Probiotic-fermented herbal blends

As a new feed additive strategy for controlling of *Salmonellae* infections, a probiotic-fermented herbal blend was recently used. The results demonstrated a significant better effect on *S. Pullorum* than an herbal mixture without probiotic fermentation (Wang et al. 2021). This is because fermenting herbs with probiotics increases the activity and extraction yield of beneficial components and the tolerance of organisms (Liu et al. 2017) as well as enhances the inflammatory responses and immunity by promoting the development of a variety of beneficial bacteria (Zhao et al. 2018). According to Junior et al. (2014), the administration of a blend of organic acids and oregano extract (acetic, formic, lactic acids, and carvacrol) either in water (0.08%) or feed (0.02%) was effective in preventing *S. Enteritidis* shedding and persistence in the broiler chickens’ crop at the slaughterhouse.

### Organic acids

Organic acids (OA) are produced in the intestines primarily through microbial fermentation of dietary carbohydrates. Their antimicrobial effects are attributed to their ability to create unfavorable conditions for pathogens, primarily by lowering the gut pH, increased proteolytic enzyme activity, increased protein digestibility, and suppresses pathogenic bacterial growth (Papatisiros et al. 2013). The undissociated form of OA is lipophilic, allowing it to diffuse across bacterial cell membranes and enter the cytoplasm, where it disrupts vital cellular functions (Dibner and Buttin 2002). The commercial OA (0.2% and 0.4%) significantly reduced viable cells from *S. Typhimurium* biofilms (Pande et al. 2018). The extent of this inhibitory effect was dependenat on product composition, exposure time, and concentration of organic acids. Acetic acid, lactic acid, benzoic acid, formic acid, and propionic acid are the most common OA incorporated in poultry rations. Numerous studies have demonstrated that dietary supplementation with OA can enhance growth performance and overall productivity in poultry. For instance, OA positively affected the morphology and physiology of the gut and enhanced immune function (Dittoe et al. 2018). Dietary inclusion of formic acid and sodium formate mixture at 0.9% was an appropriate treatment strategy to control nalidixic acid-resistant *S. Typhimurium* challenge and mantain growth performance of broiler chickens (Adhikari et al. 2020). The addition of formic acid and propionic acid (short chain fatty acids (SCFA) with or without caprylic acid and capric acid in water or feed enhanced the reduction of S. *Heidelberg* shedding and count in broilers and control the spread of S. H*eidelberg* in poultry farms as part of an adequate biosecurity program (Ferreira et al. 2022). Furthermore, the addition of formic and propionic acid succssefully controlled *S. Pullorum* in broilers and acted as a growth promoter, besides improved liver and kidney function (Khalil et al. 2022). Aljumaah et al. 2020a reported that OA supplementation improved cecal SCFAs, which could be related to the promotion of SCFA-producing microbiota, without altering major probiotic such as *Lactobacillus*. Moreover, OA supplementation modulated abnormal levels of various circulating metabolites associated with amino acid and steroid metabolism toward normal levels (Wang et al. 2019). A combination of EOs and OA was used and showed a bacteriostatic effect on *S. Enteritidis* (Zhang et al. 2019). The use of caprylic acids and trans-cinnamaldehyde acid reduced the prevalence of *S. Enteritidis* in chickens without adverse effects on the vaccinated birds (Darre et al. 2014). Nevertheless, the dietary inclusion of the OA starting at 16 weeks of age enhanced the shedding of *S. Typhimurium* in infected birds during the resurgence of infection at sexual maturity and also interfered with the oral vaccination given at 16 weeks, preventing the establishment of sufficient protective immunity (Groves et al. 2021). OA are safe antibiotic alternatives and further studies are needed to invistigate their beneficial effects and the factors affecting their outcomes on Salmonella infection and growth performamce in poultry industry.

### Probiotics

Probiotics are defined as live microorganisms that have been scientifically proven to provide significant benefits on the host (Hill et al. 2014) such as *Bifidobacterium*, *Lactobacillus*, *Bacillus* species, *Enterococcus*, and *Pediococcus* (Ruvalcaba-Gómez et al. 2022).

The dietary usage of probiotics in poultry farming is common and often preferred due to their significant contribution in inhibiting some pathogenic bacteria that pose an immense threat to poultry health (Praharaj et al. 2015). Some probiotics have been tested to prevent or control avian *Salmonella* infections at the production stages. Where the defense against intestinal bacterial infections in chicken may start with the egg, de Oliveira et al. (2014) showed that the number of *S. Enteritidis* positive chicks following oral challenge at 4 days post-hatch was significantly lower in chicks hatched from probiotics-inoculated eggs than antibiotic-fed chicks. Furthermore, the *in ovo* inoculation of a probiotic with Marek’s disease virus vaccine reduced the intestinal *S. Enteritidis* numbers and enhanced the intestinal integrity and body weight without impairing the effectiveness of the vaccine (Teague et al. 2017). Moreover, it was reported that the probiotic was as effective as the bacterin in lowering symptoms, mortality, and gross lesions, as well as in bacterial shedding and re-isolation, improving antibody titers, and improving the performance of *S. Enteritidis*-challenged broiler chickens (Abd El-Ghany et al. 2012).

Table 2 summarizes the effects of probiotic supplementation in chicken feeds to control *Salmonella* infections. The important benefits of probiotic supplementation in *Salmonella-*infected chickens are related to their ability to restore many of the microbial genera displaced by *S. Typhimurium* challenge, balance the gut microbiome, and promote a healthy gut environment (Khan and Chousalkar 2020a). In addition, probiotics may produce some antimicrobial compounds such as lactic acid, bacteriocins, hydrogen peroxide, and SCFAs which are known to inhibit the proliferation of *Salmonella* spp. (Mishra and Lambert 1996; Ruvalcaba-Gómez et al. 2022; Shaji et al. 2023). Furthermore, probiotics may compete with *Salomenella* for nutrients by sequestering essential nutrients resulting in inability of pathogens to colonize specific intestinal niches (Deriu et al. 2013). In the same line, probiotics can adhere to the intestinal mucosa and compete for the bacterial specific binding sites which inforces bacteria to leave the body before colonization. Besides, the production of organic acids may reduce the gut pH which in turn induces an unfavorable condition for the pathogens colonization (Sherman et al. 2009). Probiotics contribute to support the immune response following vaccination (Price et al. 2020; Groves et al. 2021). Probiotics can stimulate both adaptive (specific) and innate (non-specific) immunity by stimulation of immunoglobuline (IgA and IgM) secreting cells production (Ruvalcaba-Gómez et al. 2022).

**Table 2:** The different effects of probiotic supplementation in chickens to control *Salmonella* infections

Probiotic components	Experiment	Effects	Reference
Lactic acid bacteria (LAB) culture.	Day-old broiler chicks were challenged with *S. Enteritidis* or *S. Ttyphimurium* (10^4^ CFU/chick) and treated orally with LAB (4 × 10^6^ CFU/ml) an hour post-challenge.	Reduction of *Salmonella* recovery in cecal tonsils and ceca.	Higgins et al. 2007
*Lactobacillus (L.) salivarius.*	Day-old chicks were orally treated with the probiotic (10^8^ CFU/100) before *S. Eenteritidis* challenge.	Reduction of *Salmonella* in the caecal content with no recovery of the bacterium after 7days.	Kizerwetter-Swida and Binek 2009
*L. acidophilus*,* Enterococcus faecium*,* L. Plantarum*,* and L. casei.*	Day-old broiler chicks were treated with the probiotic in the drinking water for 5 days (one gm/4 liter) gainst *S. Enteritidis* challenge.	Reduction in *Salmonella* fecal shedding and and re-isolation rates from different organs.	Abd El-Ghany et al. 2012
*L. plantarum* subsp. Plantarum and *L. paracasei.*	Broiler chickens were infected with *S. Pullorum* on day 4.	Reducing mortality, balancing gut flora, promoting growth performance, and boosting immunity.	Chen et al. 2020b
*Lacticasei bacillus casei Bifidobacterium (B.) breve*,* B. Longum*,* and B. infantis*	Oral treatment with 2 × 10^9^ CFU from each probiotic bacterium against *S. Typhimurium* in broiler chickens.	Reduction of *S. Typhimurium* recovery from the cecal tonsils.	El-Sharkawy et al. 2020
*Bacillus subtilis* (KKU213, 10^8^ CFU/ml), *Pediococcus pentosaceus* (NP6, 10^12^ CFU/ml), *Pediococcus acidilactici* (SH8, 10^10^ CFU/ml*)*	After 1, 6, and 18 days of probiotic feeding, the swab samples collected from each broiler chickens’ group for the detection of *Salmonella*.	Decreased the concentration of *Salmonella* and increased the amount of lactic acid bacteria in the intestine.	Khochamit et al. 2020
*L. salivarius*,* L. gallinarum*,* L. crispatus*,* L. johnsonii*,* Bacillus amyloliquefaciens*,* and Enterococcus faecalis.*	Broiler chickens were treated with probiotic (2.0 × 10^10^ to 8.9 × 10^10^ CFU/kg feed) for 28 days. and then orally challenged with 9 × 10^7^ CFU *S. Enteritidis.*	No detection of *Salmonella* in 45% of birds at 2 weeks post-challenge.	Neveling et al. 2020
*Bacillus licheniformis*,* and Bacillus subtilis.*	Broiler chickens were orally challenged with 1 × 10^9^ CFU *S. Enteritidis*.	0.73, 1.59 and 1.32 log reduction in *Salmonella* CFU/g cecum content at 5-, 12-, and 21-days post-challenge.	Shanmugasundaram et al. 2020
*Limosilactobacillus fermentum* (PC-10 and PC-76) strains	Broiler chickens were challenged with *S. Gallinarum* (10^8^ CFU/bird) on day 21	Suppression of *S. Gallinarum*growth.	Mehmood et al. 2023
*Lactiplantibacillus plantarum (LP)* and *Lacticaseibacillus* acidophilus (LA), *Saccharomyces boulardii* (SB) and *L. delbrueckii* (LD).	Day old broiler chicks received probiotics in the feed: SB (4 × 108 CFU/Kg), LD (6 × 109 CFU/Kg), LL (3 × 109 CFU/Kg), LP (3 × 109 CFU/Kg), and LA (4.4 × 109 CFU/Kg + challenge *S. Heidelberg* at 4 day old.	Probiotic-treated broilers’ cecal content *S. Heidelberg *concentrations were noticeably lower than those of the positive control.	Hoepers et al. 2024
(Bactocell^®^ (*Pediococcus acidilactici* MA185M).	44-week-olds laying hens fed probiotic (0.1 g/kg diet) and challenged with *S. Enteritidis* by oral gavage at the start of the ninth week of the experiment.	Prior to and following the *S. Enteritidis* challenge, probiotics had a stronger impact in lowering blood cholesterol levels and yolk cholesterol, respectively. It reduced cecal *S.Enteritidis* colonization post challenge.	Kalani et al. 2022
*Lactobacillus spp.*	Domestic chickens (Bábolna Tetra-SL laying hybrid breed) fed probiotic at 1 kg/T and were infected with mixed infection with *S. Enteritidis* and *E. coli* by oral gavage on day 3 of age.	İt significantly reduced Salmonella shedding compared to enrofloxacin-treated group.	Kerek et al. 2023
*L. acidophilus*,* Lacticasei bacillus rhamnosus GG (LGG)*,* or B. animalis subsp. lactis (Bb12).*	One-day-old SPF leghorn chickens received LA, LGG, or Bb12 daily using oral gavage from day 1 until day 16 of age. + *S. Typhimurium* challenge on day 7.	LGG exhibited superior probiotics properties and significantly reduced the *S. Typhimurium* load and improved intestinal histomorphology. Also, LGG increased abundance of beneficial *Bacillis*,* Butyricicoccus*,* Erysipelatoclostridium*,* Flavonifractor.*	Closs et al. 2025

Compared to live probiotics, inactivated probiotics (non-viable microbial cells), often known as paraprobiotics or ghost probiotics, have demonstrated health benefits (de Almada et al. 2016). Heat stressed-broiler chickens and challenged with *S. Enteritidis* showed increased live weight and feed efficiency following feed supplementation with yeast protein concentrate and pellets (Haldar et al. 2011). These benefits were numerically larger than those that were achieved with *Saccharomyces Cerevisiae* supplementation. Therefore, the use of probiotics as dietary supplements can lead to improvement in growth performance and reduce the negative influences of salmonellosis on poultry health.

### Prebiotics

Prebiotics are nondigestible carbohydrates including oligosachharides (galacto-oligosaccharides (GOS), mannan-oligosaccharides (MOS), and fructo-oligosaccharides (FOS) that are utilized by the gut beneficial bacteria. These compounds provide the essential energy necessary for the optimal growth and proliferation of the naturally beneficial bacteria. Moreover, prebiotics inhibit the colonization and extension of detrimental bacteria such as *E. Coli*, *Campylobacter Jejuni*, and *Salmonella* spp. and thus help to beneficially regulate the complex ecosystem of the gut microbiota. Therefore, they create healthy intestinal flora for the regular functioning of the digestive system and strengthening the immune system (Awaad et al. 2013; Ricke 2018). Compared to antibiotics, prebiotic components can be utilized more efficiently and safely.

According to Shang et al. 2015; a major impact of *S. Enteritidis* lipopolysaccharides on the immune responses of broiler chickens was observed in an immunological challenge experiment. They reported that the broiler chickens’ intestinal morphology and innate and humoral immunological responses were protected by dietary FOS supplementation, without compromising their growth performance. Moreover, laying hens given 0.5% or 1% FOS demonstrated a significant decrease of the caecal load and fecal shedding, an increase in IgA + cells in the ileal lamina propria, and up-regulation of TLR-4 and IFN-γ expression following *S. Enteritidis* infection (Adhikari et al., 2018). The different effects of prebiotics against *Salmonella* infections in poultry are shown in Table 3. These observable variable effects are contingent upon the prebiotic’s constituents, concentration, dosage, and delivery time. According to a systematic review and meta-analysis of Malli et al. 2025, probiotics and prebiotics both lessen *Salmonella* colonization and shedding in the feces and cloacae of broiler hens, although the benefits of prebiotics, particularly MOS, are marginally more pronounced.

**Table 3:** The different effects of prebiotics against *Salmonella* infections in poultry

Prebiotic components	Experiment	Results	Reference
Fructooligosaccharide (FOS) at 0.75% level in the feed.	Broiler chickens were stressed by feed and water deprivation on day 13 and orally challenged with 10^9^ *S. Typhimurium* on day 14.	33 of 36 (92%) chickens fed a control diet were colonized compared with only 9 of 36 (25%) chickens fed a 0.75% FOS diet.	Bailey et al. 1991
Mannose oligosaccharides (MOS) (Derived from *Saccharomyces Cerevisiae* cell wall) (4.000 ppm in feed).	Experiment 1: Broiler chickens were orally challenged with 10^4^ CFU of *S. Typhimurium* on day 3 Experiment 2: Broiler chickens were orally challenged with 10^4^ CFU of *S. Dublin* on day 3.	Reduced the cecal *S. Typhimurium* concentrations in chickens fed with MOS (4.01 vs. 5.40 log CFU/g) 7 days post- challenge. Fewer birds tested positive for *S. Dublin* (56 vs. 90%) in the ceca on day 10 of MOS supplementation.	Spring et al. 2000
Bio MOS™: 2 kg/ton up to 10 days; 1 kg/ton (10 to 21 days; and 0.5 kg/ton: 21–28 days.	Broiler chickens were orally challenged with *S. Enteritidis* (10^6^ CFU) on day 3 of age.	No influence on performance or production but resulted in lower cecal SE count than Avilamycin-treated group.	Ribeiro et al. 2007
Inulin, FOS, MOS, and oligosaccharide.	Day-old layers and broilers chicks were challenged with *S. Enteritidis.*	The protective effect of prebiotics was determined during the first week after *Salmonella* infection.	Murate et al. 2015
MOS	Seven-days-old turkey poults were orally challenged with 10^5^ CFU/ml *S. Heidelberg.*	Reduced *Salmonella* shedding.	Rahimi et al. 2019
Beta LevaFructan™	Each broiler chicken was orally challenged with 0.5 ml suspension containing 10^9^ CFU/ml *S. Enteritidis.*	The use of prebiotics simultaneously with live *Salmonella* vaccine administration may be preferable to reduce the negative impact of the bacteria and improve the growth performance.	El-Shall et al. 2019
Diamond V Original XPC™ (post fermentation growth medium residues, residual yeast cells, and β-glucans, and MOS).	44-week-olds laying hens fed prebiotic (1.25 g/kg diet) and challenged with *S. Enteritidis* by oral gavage at the start of the ninth week of the experiment.	Prior to and following the *S. Eenteritidis* challenge, prebiotics had a stronger impact in lowering blood cholesterol levels and yolk cholesterol, respectively. It significantly reduced *S. Eenteritidis* colonization in the ceca post challenge.	Kalani et al. 2022

Therefore, prebiotics can increase the host’s resistance to pathogens and prevent the invasive bacteria from adhering to intestinal mucosa. They are broken down by certain species of intestinal flora and used as metabolic substrates or sources of energy (Kaplan and Hutkins, 2000). The previous studies have described the hydrolysis and utilization of prebiotics by probiotic bacteria (Cecchini et al. 2013). Consequently, it is highly probable that a multitude of gut microbiota plays a significant role in the complex process of prebiotic metabolism, thereby adding layers of intricacy to our comprehension of their profound effects on the host organism as well as their inhibitory influence on pathogens that are detrimental to the host’s well-being. For instance, fermentation of prebiotics by intestinal microflora results in volatile fatty acides such as butyrate that reduces intestinal pH and promotes the proliferation of intestinal epithelial cells (Bogusławska-Tryk et al. 2021). In addition, it is possible that other interactions may occur independently of the gut microbiota, suggesting that the relationship between prebiotics, microbiota, and pathogens is even more multifaceted than previously thought (Alloui et al. 2013; Micciche et al. 2018).

### Synbiotics

The approach of combining probiotic and prebiotic ingredients, known as synbiotic intervention, has been studied and showed a number of positive benefits on chicken against *Salmonella* and other enteric infections. Synbiotics provide the substrates essential for probiotic fermentation and thus promote their growth (Hassanpour et al. 2013). Therefore, synergestic effects were noticed in chickens’ growth performance, carcass traits (Awad et al. 2009), and intasetinal histomorphology (Raksasiri et al. 2018). Moreove, synbiotic products increased level of protection and inhibitory impact on *S. Typhimurium* organ invasion and cecal colonization in broiler chicks (Revolledo et al. 2009 and improved secretory IgA and intestinal microbiota in *S. Enteritidis*-challenged laying hens (Markazi et al. 2018).

The combinations could contain probiotic, prebiotic, organic acids, enzymes, and/or postbiotic. Jazi et al. 2018 compared the efficacy of dietary inclusion of MOS, Pediococcus acidilactici, butyric acid, and their combination on intestinal health and growth performance in three day old broiler chicks challenged with *S. Typhimurium*. According to their findings, the combination of the three additions exhibited the highest weight increase, feed intake, and populations of cecal *Lactobacillus* and *Bifidobacterium*, while having the lowest populations of cecal *Salmonella* and *Coliforms* and the heterophil to lymphocyte ratio. Additionally, synergistic effects were achieved both in vitro and in vivo by combining a dietary prebiotic (inulin) and a postbiotic (metabolic products of lactic acid bacteria). In vitro, they have antimicrobial effects on a variety of pathogenic bacteria, including *S. enterica* (Kareem et al. 2014). In broiler chickens, they improved the intestinal mucosa architecture, growth performance, expression of IGF1 and GHR mRNA, and the number of lactic acid bacteria in droppings, while decreased the number of *Enterobacteriaceae* (Kareem et al. 2016). Table 4 shows the different effects of synbiotics on *Salmonella* infection in chickens.

**Table 4:** The different effects of synbiotics on *Salmonella*-infected broiler and layer chickens

Synbiotics components	Experiment	Main effects	Reference
85% (*Bacillus subtilis*,* L. acidophilus*,* L. casei*,* Enterococcus faecium*,* Bifidobacterium*) and 15% (inulin, FOS, MOS, and oligosaccharide).	1-day-old layers and broilers chicks were challenged with *S. Enteritidis*, and synbioticwas added to the ration as feed additives.	Synbiotic showed limited ability to reduce *S. Enteritidis* colonization.	Murate et al. 2015
Prebiotic besides *Saccharomyces* sp., and *Lactobacillus* sp.	21-day-old Cobb broiler chicks were maintained for 36 days and challenged with *S*. E*nteritidis*. Synbiotic was incorporated into the diet.	Feed additive increased weight gain and maintaind immunity as shown in the blood test values.	Ajiguna et al. 2021
FOS, *Pediococcus* acidilactici, Bifidobacterium animalis, *Enterococcus faecium*, and *Lactobacillus reuteri.*	At 21 days of age, they were orally challenged with *S. Typhimurium.* Chicks were fed synbiotic at 1 g/kg of feed starting from day one.	The addition of synbiotic reduced Salmonella colonization in the liver and induced marked changes in the expression of proinflammatory cytokines.	Elsharkawy et al. 2024
Inulin, *Lactobacillus rhamnosus* and *Pediococcus acidilactici*.	At 17 days of age, broiler chicks challenged orally with *S. Typhimurium* received the synbiotic mix via drinking water.	The broiler’s ability to resist infection is attributed to morphological changes, including longer villi and shallower crypts.	Villagrán-de et al., 2019
*L*. *reuteri*, *E*. *faecium*, *B*. *animalis*, *P*. *acidilactici* and *a* FOS	At 21 days of age, broiler chicks challenged orally with *S. Enteritidis* and were administered the synbiotic mix as a feed additive.	Reduce Salmonella colonization in the cecal contents.	Shanmugasundaram et al. 2019
*B. subtilis*,* B. licheniformis*,* and S. cerevisiae*	At 21 days of age, broiler chicks challenged with *S. Typhimurium* and received the synbiotic as a feed additive.	Synbiotic increased feed intake improved intestinal morphology, increased IgG and IgM levels, and enhanced the expression of immune-related genes.	Fazelnia et al. 2021
Inulin, *L. rhamnosus, and Pediococcus acidilactici.*	At 17 days of age, broiler chicks were challenged with *S. Typhimurium* via drinking water. The synbiotic was administered in the drinking water for 2 h daily, starting from the first day of life.	Synbiotic increased the size of follicles of the bursa of Fabricius, boosting IgA production, and enhanced the broiler’s ability to resist *S. Typhimurium* infection.	Villagrán-de et al. 2020
Inulin, *Enterococcus faecium*, *Pediococcus acidilactici*, *B. animalis*, *Limosilactobacillus reuteri*, and *Ligilactobacillus salivarius*	On day 7 of age, broiler chicks challenged with *S. Infantis* and treated with synbiotics, in the drinking water, feed, or both.	Synbiotic reduced *S. Infantis* colonization in the cecum, ileum, and spleen.	Drauch et al. 2025
Fulfill Plus™ (*Bacillus subtilis*, B. Licheniformis, and MOS).	Day old-Hy-line pullets were treated with Fulfill Plus at 1.5 lbs/ton (0.075%) at day 0 or week 10 to week 17. Then, at 17 weeks of age, a 3 × 10^6^ CFU/bird nalidixic acid-resistant strain of *S. Enteritidis* was given orally.	Significant reduction in *S. Enteritidis* number in the ceca.	Kimminau et al. 2021
Synbiotic A: *Enterococcus sp.*,* Pediococcus sp.*,* Bifidobacterium sp.*,* Lactobacillus sp*. and FOS. Synbiotic B: *L. acidophilus*,* L. casei*,* L. salivarius*,* L. plantarum*,* L. rhamnosus*,* L. brevis*,* B. bifidum*,* B. lactis*,* S. thermophiles*, prebiotic inulin, protease, amylase, cellulase, hemicellulase, lipase, papain and bromelain.	At 8 days of age, layer chicks were challenged via the oral route with S.Typhimurium. The synbiotic mix was added to drinking water or ration from the day of hatch until the 7-day.	Synbiotics increased the abundance of several beneficial bacterial genera in infected chicks. But they were not effective in reducing *S. Typhimurium* load in the liver, spleen, or cecal tissues.	Khan and Chousalkar 2020b
*Bacillus subtilis* and *Saccharomyces cerevisiae* cell wall.	At 8 days of age of Lohmann LSL layer chicks were challenged with *S. Enteritidis*, and the synbiotic was added as feed additive.	Synbiotic reduced concentration of *S. Enteritidis* in the ceca.	Araba et al. 2024
*Bacillus subtilis* and yeast cell wall-derived glucomannan.	At 21 days of age of layer chicks were challenged with *S. Enteritidis* via the oral route and synbiotic was administered as a feed additive.	Synbiotic reduced the cecal Salmonella prevalence, thereby lowering public health risk and supporting food safety programs.	Girgis et al. 2022
PoultryStar^®^ (*Lactobacillus reuteri*,* Bifidobacterium animalis*,* Pediococcus acidilactici*,* Enterococcus faecium*,* and FOS)*	One-day-old layer chicks received synbiotic product in drinking water and challenged with *S. Enteritidis* at 24 weeks of age.	76.0% increase in biliary anti-Salmonella IgA, a significant improvement in intestinal microbiota, and a decrease in LITAF mRNA expression were observed.	Markazi et al. 2018

### Postbiotics

Postbiotics are defined as non-living bacterial metabolites or metabolic products derived from microorganisms with biological activities in the host (Martín and Langella 2019) or secreted following bacterial lysis (Aguilar-Toal et al. 2018). Numerous bacterial strains, primarily *Bifidobacterium*, *Lactobacillus*, *Streptococcus*, and *Faecalibacterium* spp., can produce postbiotic metabolites, which include organic acids, teichoic acids, SCFAs, peptides, plasmalogens, enzymes, vitamins, polysaccharides, peptidoglycan-derived muropeptides, or cell surface proteins (Oberg et al. 2011; Tsilingiri and Rescigno 2013). Therefore, the primary constituents of postbiotics are OA and peptides. The microbiota recognizes these molecules and uses them to control gene expression, ensuring the survival and collective modulation of microorganisms that promote health (Abisado et al. 2018). Postbiotics can aid in the stabilization of the gut microbiome of chickens and promotion of both specific and non-specific immune responses in the host (Abd El-Ghany et al. 2022). The soluble metabolites produced by lactic acid bacteria have proven to be equally effective as antibiotics in significant enhancement of growth performance of broiler chickens when utilized as feed additives (Humam et al. 2021). Postbiotics showed broad antimicrobial activity against Gram-positive and -negative bacteria in vitro (Kareem et al. 2014). Postbiotic-*Lactobacillus* bacteriocins, rich in arginine, lysine, and histidine, have cationic characters in a neutral pH conditions, which enable them to bind pathogens and disrub their cellular integrity and conseuently decreases the clinical picture associted with *Salmonella* infections (Ruvalcaba-Gómez et al. 2022; Shaji et al. 2023). Moreover, postbiotic treatment could induce the production of Albusin B, increase the expression of endothelin 1 precursor protein in the jejunum of broiler chicks, enhance intestinal glucose and protein absorption, and inhibit *Salmonella* colonization using its lectin structural domain (Ruvalcaba-Gómez et al. 2022; Shaji et al. 2023). Table 5 shows the strategic implications of postbiotic supplementation for *Salmonella* control in poultry.

**Table 5:** Strategic implications of postbiotics supplementation for *Salmonella* control in poultry

Postbiotic (PB)	PB application	Chickens/Salmonella challenge	Main results	Reference
*Saccharomyces Cerevisiae* fermentation–based PB.	PB was added to starter diet by 1.5 kg/metric ton (MT) (from day 0 to day 21) and to the grower diet by 1.0 kg/MT (from day 22 to day 32 (end)).	Hy-Line W-36 layer pullets were challenged with *S. Eenteritidis* (1 × 10^6^ CFU) on day 28.	The concentration of *S. Eenteritidis* was lower in postbiotic group than control. Postbiotic-fed pullets had a lower percentage of birds with enumerable *S. Eenteritidis* concentrations (57.9%) than non-treated pullets (95.0%).	Gingerich et al. 2021
*Saccharomyces cerevisiae-*derived PB.	PB was added by 1.25 kg/MT from day 0 to the market age (33–34 days).	12 houses of a commercial broiler chicken (day of hatch Cobb 500 or Ross 308) farm/no challenge.	Litter prevalence for Salmonella were equivelant, while ceal prevalence was lower in PB-treated than controls. The estimated burden of PB-treated flocks was lower (3.80 log_10_ MPN) vs. control (7.31 log10 MPN).	Chaney et al. 2022
Diamond V, Original XPC^®^ *(Saccharomyces Cerevisiae* fermentation-derived PB).	2.5 lb./ton PB was fed starting from day 0.	Day-old W-36 layer chicks. At 16 weeks, pullets were orally challenged with SE resistant to nalidixic acid strain (1.1 × 10^9^ CFU). Plus, contact chickens exposed to horizontal transmission.	* S. Eenteritidis* prevalence in the ovaries and ceca of directly challenged birds did not significantly differ between groups. The contact birds showed considerably lower *S. Eenteritidis* cecal load (1.69 Log10) vs. non-treated (2.83 Log10) and lower *S. Eenteritidis* cecal prevalence at 7 dpi for treated (50.0%) compared to non-treated (72.5%) group.	Chaney et al. 2023
*Lactobacillus Helveticus* ATCC 15,009-derived PB.	PB inoculum given to chicks on the 19th day of age by oral gavage.	Laying chicks (Hy-Line W80) were challenged with *S. Gallinarum* (10^9^ CFU/ml) on day 21 by oral gavage.	Reduced count of *S. Gallinarum* in the cecum and liver of chicks and modulated the intestinal microbiota.	Ribeiro et al. 2023
*Lactiplantibacillus Plantarum*-derived PB.	0.8% PB in drinking water for 28 days.	One-day-old yellow-feathered broilers/challenge with *S. Eenteritidis* (1 × 10^9^ CFU) on day 28 by oral gavage.	PB maintained the growth performance of infected chicks. It provided protection against intestinal damage via suppressing NLRP3 inflammasome and modulating gut microbiota.	Guan et al. 2024
*Bifidobacterium Bifidum* PB.	1 mL of PB daily via oral gavage.	1-day-old Partridge Shank chicks/oral challenge with *S. Pullorum* at 7 days old.	PB reduced mortality from 66.66% to 8.33%, and considerable improved growth performance. BbP decreased inflammatory cytokines (IL-1β, IL8) and decreased pyroptosis-related protein expression while raising anti-inflammatory cytokines (IL-10, IL-4).PBO restored intestinal barrier function and improved cecal microbiota diversity.	Chen et al. 2025

### Nanoparticles

Nanoparticles can be produced in the laboratory utilizing both conventional chemical and physical techniques as well as environmentally friendly and sustainable processes like biosynthesis. Nanoparticles’ biological characteristics and their impact on animal performance and health have received particular interest (Michalak et al. 2022; Abd El-Ghany 2019). Nanoparticles are becoming widely used with vaccines and in the diet as food supplements due to their high absorption in the body. They can be transported directly to target organs and prevent the rapid degradation that can occur with antibiotics (Gangadoo et al. 2016).

Several metal nanoparticles, nanoemulsions, and nano-encapsulated essential oils have been investigated for their antimicrobial activities against *Salmonella* spp.

In vitro, the gold nanoparticles showed higher antibacterial activity for *S. Typhi* and *S. Paratyphi* of food origin than ciprofloxacin, indicating an alternative use of chemically synthesized gold nanoparticles in biomedicine (Boatemaa et al. 2019). In addition, a potential antibacterial efficacy against MDR *S. Eenteritidis* and *S. Typhimurium* isolates was demonstrated by biosynthesized silver nanoparticles (AgNPs). AgNPs exhibited a minimum bactericidal concentration of 6 and 8 µg/mL against isolates of *S. Enteritidis* and *S. Typhimurium*, respectively, and a minimum inhibitory concentration of 5 µg/mL against these isolates (Abou Elez et al. 2021). Moeover, AgNPs demonstrated significant antibacterial activity (inhibit 60–90% of S. *Typhimurium)* that was more pronounced at low concentrations (Gabrielyan et al. 2020). Zinc oxide nanoparticles (Akbar and Anal 2014) and citric acid coated-iron oxide nanoparticles (Gabrielyan et al. 2020) showed efficacy in inhibiting *S. Typhimurium* growth. In addition, water-in-oil nanoemulsions, which are made up of droplets that range in diameter from 100 to 1000 nm (Alliod et al. 2018), can preferentially fuse with bacterial cell walls, disturbing the lipid of the organism and causing disruption to the pathogen (Baker et al. 2003). They are able to accomplish this without causing harm to the eukaryotic cells in the tissue (Chepurnov et al. 2003) or creating resistant strains (Ramalingam et al. 2010). Joe et al. 2012 reported that sunflower oil-based nano-emulsion formulation (72.52 to 875.22 nm) showed high antimicrobial activity against *S. Typhi* using disc inhibition assay.

In vivo, the quantity of beneficial intestinal microbiota was enhanced in rainbow rooster chickens when exposed to garlic and onion extract-chitosan nanoparticles, whereas harmful enteric bacteria such as *S. Typhi*,* C. Jejuni*, and *E. Coli* showed limited colonization (Enoka et al. 2021). The supplementation of broiler diets with 0.5% and 1% thymol nano-emulsion could reduce *S. Typhimurium* populations, down-regulate *invA* gene expression after infection, and increase the beneficial intestinal *Lactobacilli* counts (Ibrahim et al. 2021b). In comparison to the control and ciprofloxacin-treated groups, Nassar et al. (2025) found that oral gavage of thyme oil-loaded chitosan nanoparticles significantly decreased *S. Typhimurium*-induced postmortem lesions in broilers and lessened the systemic and localized inflammatory response. These effects also were superior to individual treatments (chitosan NPs or thyme oil). They recommnded thyme oil-loaded-chitosan naoparticles as an alternative antibacterial agent in controlling *S. Typhimurium* infection without taking the risk of developing resistant bacterial strains as with antibiotics. Neverethless, there is a need for more research into the practical use of nanotechnology products in poultry farms and generalization in poultry systems in order to create safe, affordable, and environmentally friendly substitutes for antibiotics.

### Egg yolk immunoglobulin Y

As a result of the interplay between innate and specific immunity, the body produces specialized proteins known as immunoglobulins to fight diseases and respond to them in a specific way. These molecules’ capacity to attach antigens selectively makes them useful for both therapeutic and diagnostic purposes. Recently, hyper-immunization of laying hens for isolating egg yolk IgY has become a popular technique for producing huge amounts of antibodies (Han et al. 2021; El-Kafrawy et al. 2023). The egg yolk-extracted IgY can be given to chickens orally for immunotherapeutic purposes. Using immunofluorescence and immunoelectron microscopy, *Salmonella*-specific IgY was found to be able to attach to the antigens produced on the *Salmonella* surface, which caused structural changes to the bacterial surface (Lee et al. 2002). According to Lee et al. (2002), *Salmonella* growth in liquid media was inhabited in vitro by egg yolk IgY specific to *S. Enteritidis* and *S. Typhimurium*. In comparison to one another, they showed cross-reactivity of 55.3% and 42.4%, respectively. The administration of specific anti *S. Enteritidis* or anti S. *Typhimurium* polyclonal IgY to *Salmonella* infected chicks reduced the bacterial fecal load by up to 40% (Karabasanavar et al. 2022). The colonization of the liver and cecum of broilers with *S. Infantis* was significantly reduced after administration of encapsulated specific IgY in comparison with other types of antibodies (Isfahani et al. 2020). In addition, *S. Enteritidis-*specific IgY combined with probiotic containing *Lactobacillus*,* Enterococcus*, and *Bifidobacterium* spp. successfully reduced *Salmonella* colonization in broilers (Rahimi et al. 2007). The study of Li et al. (2016) showed that chicken IgY reduced the jejunum damage induced by *S. Typhimurium* infection in mice, besides, the reduction in cytokines (IFN-γ and TNF-α) levels and modulation of intestinal mucosal immune responses. These studies demonstrate the potential effectiveness of passive immunity, or egg yolk antibodies, in preventing *Salmonella* infections and possessing antibacterial properities. Future studies can investigate the feasibility of producing IgY on a large scale for use in poultry systems.

### Bacteriophage therapy

Bacteriophages, as a substitute for antibiotics, can target specific bacteria and help lowering the *Salmonella* counts. If an appropriate phage is discovered or genetically modified phages are created, even pan-antimicrobial resistant bacteria can be made vulnerable to phage attack (Khan and Rahman 2022). Bacteriophages are viruses that infect bacteria in two different ways (lytic and lysogenic) at the genus or species level (Kasman and Porter 2022). The lytic phages cause cell lysis and release of new phages into the environment after replication inside the bacterial cell (lytic cycle). Therefore, lytic phages are preferred for therapeutic purposes to eliminate pathogenic bacteria. Nevertheless, lysogenic phages integrate into the host cell genome, the so-called lysogenic cycle, and become prophages. The prophages remain immobilized until they are activated, at which point it replicates and begins a new lytic cycle. Some phages can exhibit both lytic and lysogenic behaviors, depending on the environmental conditions and the specific phage-host interaction (Sulakvelidze et al. 2001; Khan and Rahman 2022).

It was noted that oral phage treatment in broilers dramatically decreased *Salmonella* colonization, which was crucial in ending the cycle of transmission from parent hens to chicks (Sonalika et al. 2020). Phage therapy is effective depending on factors such as the selection of appropriate phages, dosage, method of administration, and appropriate duration of treatment (Wernicki et al. 2017). Prepared phage solutions can be administered orally (by adding to feed or drinking water), sprayed on eggs, or directly added to contaminated products to prevent horizontal and vertical transmission of *Salmonellae.* The lytic phage, Felix-O1 is preferred in a number of therapeutic and diagnostic applications because of its capacity to affect distinct *Salmonella* serovars (Carvalho et al. 2012). Table 6 summarizes the results of the different experimental studies utilizing bacteriophage preparations in poultry.

**Table 6:** Phage therapy against Salmonella infections in chickens

Birds	Salmonella challenge	Phage	Phage application	Main results	Reference
Broiler chickens	*S. Enteritidis*,* S. Hadar *,* S. İnfantis *,* S. Typhimurium *, and *S. Virchow * at 7 log CFU/mL. Oral gavage on day 17.	Phage cocktail: (vB_SenS_KP001, vB_SenS_KP005, and vB_SenS_WP110. *S. Anatum* A4-525).	Oral gavage of a daily dose of the phage cocktail at 10 log PFU/mL, from day 1 to 16 (prevention group) and 18 to 31 (exclusion group).	Prevention and exclusion programs may control Salmonella on days 20 and 32 of the trial, respectively. Salmonella was not detected in cloacal swabs (0% prevalence) 16 days afterphage treatment.	Pelyuntha et al. 2024
Seven-day- old chickens	* S. Enteritidis* at 2.95 × 10^5^ CFU/ml on day 7.	Three Salmonella-specific phages in combination with a competitive exclusion compound (CE).	Phage mixture (10^3^ PFU) and CE were administered by spray 24 h prior to Salmonella challenge at 1 day of age.	The incidence of *S. Enteritidis* decreased to 75.7% with CE and to 80% with phage mixture; when CE and phage were used, the incidence decreased to 38.7%, compared to the control group (100%).	Borie et al. 2009
Broiler chickens	No challenge.	Three Salmonella phage: (vB_SenS_KP001, vB_SenS_KP005, and vB_SenS_WP110)	9 log PFU/mLof phage was adminstered orally using a feeding tube, and nipple waterer (for 30 min drinking time). Five doses on days 0, 4, 13, 16, and 20	Phage dramatically reuced intestinal Salmonella colonization. The Salmonella prevalence was decreased from 70% to 0% after 4 days of phage treatment.	Pelyuntha et al. 2022
Six-week- old commercial layer chickens	*S. Gallinarum* (5 × 10^8^).	*S. Ggallinarum*-specific phage.	Contact chickens given 10^6^ PFU/kg of phage as feed additives for 7 days before, and 21 days following challenge.	Contact chicken treated with the phage showed a significant drop in mortality and *S. Ggallinarum* reisolation from organs, compared to untreated contacts.	Lim et al. 2011
White Leghorn chicken specific-pathogen-free (SPF) chickens	0.1 ml of *S. Typhimurium* / Oral.	Three phage: UAB_Phi20, UAB_Phi78, and UAB_Phi87.	Oral administration of 0.1 ml of the phage cocktail at 10^11^ PFU/ml.	When the phage cocktail was given one day prior to or immediately following *S. Typhimurium* infection, and then again on various days after infection, effectively decreased *S. Typhimurium* concentration in the cecum.	Bardina et al. 2012
One-day-old chicks	5 × 10^7^ CFU/bird of *S. Enteritidis* and were cohabitated with contact chicks.	ΦCJ07.	Three concentrations of phage (10^5^, 10^7^ and 10^9^ PFU/g). Orally (feed additive) for 21 days post challenge.	Intestinal *S. Enteritidis* colonization was decreased in challenged and contact chickens by al phagel , especially by 10^7^ and 10^9^ PFU Three weeks following therapy, phage at 10^9^ PFU/g resulted in 70% of contact chickens were negative for SE detection.	Lim et al. 2012
SPF chicks	0.25 ml of 10^10^ CFU/ml of ST/bird.	* S. Typhimurium* lytic bacteriophage (Φ st1).	0.25 ml of 10^12^ PFU/ml Φ st1 following challenge/Intra-cloacal.	Within 6 h of the challenge, the *S. Typhimurium* count was decreased in cloacal swabs and visceral organs. No *S. Typhimurium* was found at or after 24 h post therapy.	Wong et al. 2014
One day old SPF chicks	* S. Typhimurium* and *S. Enteritidis* individually (10^5^ CFUs) at day 2 orally.	* S. Typhimurium* and *S. Enteritidis* phages.	1.18 × 10^11^ PFU of *S. Typhimurium* phage/chick and 1.03 × 10^12^ PFU of *S. Enteritidis* phage/chick orally at days 1, 2, 3, 6, 8, 10, 13 and 15.	Salmonella loads in cecal contents decreased slightly at 3 days post-infection (dpi), and more significantly at 5 dpi. From 7 dpi to the experiment’s conclusion at 15 dpi, every chick tested negative for both Salmonella. Cecal Salmonella colonization drop was improved after five phage doses.	Nabil et al. 2018
Commercial broiler chickens	No challenge.	Phage cocktail SalmoFREE^®^.	10^8^ PFU/mL in the drinking water at 3 time points in the production cycle.	Reduced *Salmonella* incidence without negative effects on the production metrics.	Clavijo et al. 2019
One day old- broiler chickens	* S. Enteritidis* PT4.	Three-phage cocktail: CNPSA1, CNPSA3 and CNPSA4.	10^11^ PFU of each of three phage orally administered on the 7th day of age.	Cecal *S. Enteritidis* count decreased by 3.5 orders of magnitude in the phage-treated group five days after therapy and continued to reduce on days 10, 15, 20, and 25 after treatment.	Fiorentin et al. 2005
Broiler chickens	10^5^ CFU *S. Enteritidis*/g feed from day 8 to 14.	* S. Enteritidis* specific phage.	10^8^ PFU phage in the feed (1 and 1.5 kg/metric ton).	* S. Enteritidis* prevalence was decreased by both phage treatments in cecum on day 28 and cloacal swabs on day 14. On day 28, *S. Enteritidis* prevalence in liver and spleen did not differ significantly.	Kimminau et al. 2020
22 weeks old- adult broiler breeders	1.8 × 10^9^ CFU of *S. Pullorum* by injection into the breast muscle.	*Salmonella* phage CKT1.	Single dose of phage (1 × 10^8^ PFU/chicken) at 3 weeks post infection orally.	The phage reduced *S. Pullorum* load in liver, spleen, heart, ovary, and oviduct and mitigated hepatic damage. It decreased serum of Salmonella-specific IgG and the vertical transmission of *S. Pullorum* from chickens to laid eggs.	Cui et al. 2023
Broiler chickens	10^6^ CFU/bird of *S. Typhimurium* SL1344 strain at day 4 by oral gavage.	Phage cocktail: Phages SPFM10 and SPFM14.	10^5^, 10^6^, 10^7^ PFU/day of phage in mash diet throughout the study (42 days).	enhanced weight increases and improved growth performance in challenged birds as compared to challenged birds not fed phage.	Thanki et al. 2023
Newly hatched chicks	5 × 10^4^ CFU/chick of *S. Enteritidis* at 7 days of age.	Six-phages cocktail.	10^9^ PFU/mL phage via drinking water for 6 days after hatching.	Cecal *S. Enteritidis* colonization decreased dramatically by 3 log up to 4 dpi. However, 8 days after stopping phage treatment, Salmonella levels returned. Dysbiosis was not induced by the phage cocktail.	Agapé et al. 2024
Broiler chickens	* S. Enteritidis* orally.	Salmonella phage (vB_SalS_JNS02).	10^8^ PFU phage administered orally 3 h after *S. Enteritidis* infection (single dose).	Phage successfully shielded from *S. Enteritidis* infection. The relative abundance of beneficial bacteria rose along with the production of secretory IgA.	Li et al. 2024
34 day old- broiler chickens	*S. Enteritidis*, *S. Hadar*, and *S. Typhimurium*, separatly on day 36 of age by oral gavage.	Three Salmonella phages: Φ151 (*S. Enteritidis* P125109), Φ25 (*S. Hadar* 18), or Φ10 (*S. Typhimurium* 4/74).	Three Salmonella phages: 10^9–11^ PFU by oral gavage 2 days post salmonella infection.	Within 24 h, phage decreased *S. Typhimurium* and *S. Enteritidis* cecal colonization by ≥ 2.9 log10 CFU and ≥ 4.2 log10 CFU, respectively. *S. Hadar* colonization was not decreased by the third phage.	Atterbury et al. 2007

Bacteriophages offer several unique advantageous features, making them a suitable alternative to antibiotics. Phages have a high degree of host-specificity in contrast to antibiotics, which lowers the risk of intestinal dysbiosis and subsequent infection after phage therapy. The development of a new antibiotic is a costly and time-consuming process. In contrast, the isolation, replication, and large-scale production of phages are less expensive. Moreover, they can spread throughout the body during systemic administration, cross the blood-brain barrier, and inhibit biofilm formation (Azeredo and Sutherland 2008; Wittebole et al. 2014). Remarkably, phage therapy-resistant bacteria are thought to be less important than antibiotic-resistant bacteria. Given that phages are genetically adjustable, current genetic engineering techniques can be used to counteract any phage resistance that the bacteria may develop. The utilization of phages is acknowledged as a safe, natural, viable, and cost-effective method for *Salmonella* control in poultry before and after slaughter (Torres-Acosta et al. 2019).

## Conclusion

Various *Salmonella* species continue to spread among chicken flocks resulting in losses and zoonotic risk. In addition, the development of MDR *salmonella* species and risk of environmental resideus continue to surface on th scene. The sustainable natural substitutes therefore lessen the need for antibiotics; meeting demands for safe and organic chicken production methods. These interventions can lower mortality, postmortem lesions, the intestinal *Salmonella* count and shedding rate, enhance the balanced gut flora, and boost growth performance and immunity. Ongoing research should be conducted to investigate the safety margin, spectrum of activity on *Salmonella* species, and the practical methods of application for these measures in the field.

## Data Availability

The Data supporting this study can be provided by the corresponding author whenever it is needed.
